# Proficiency of Extracellular Vesicles From hiPSC-Derived Neural Stem Cells in Modulating Proinflammatory Human Microglia: Role of Pentraxin-3 and miRNA-21-5p

**DOI:** 10.3389/fnmol.2022.845542

**Published:** 2022-05-17

**Authors:** Raghavendra Upadhya, Leelavathi N. Madhu, Shama Rao, Ashok K. Shetty

**Affiliations:** Department of Molecular and Cellular Medicine, Institute for Regenerative Medicine, Texas A&M University College of Medicine, College Station, TX, United States

**Keywords:** antiinflammatory effects, extracellular vesicles, microglia, human induced pluripotent stem (hiPS) cells, human neural stem cells, lipopolysaccharide, neuroinflammatioin

## Abstract

Extracellular vesicles (EVs) shed by human-induced pluripotent stem cell (hiPSC)-derived neural stem cells (hNSC-EVs) have shown potent antiinflammatory properties in a mouse macrophage assay and a mouse model of acute neuroinflammation. They can also quickly permeate the entire brain after intranasal administration, making them attractive as an autologous or allogeneic off-the-shelf product for treating neurodegenerative diseases. However, their ability to modulate activated human microglia and specific proteins and miRNAs mediating antiinflammatory effects of hNSC-EVs are unknown. We investigated the proficiency of hNSC-EVs to modulate activated human microglia and probed the role of the protein pentraxin 3 (PTX3) and the miRNA miR-21-5p within hNSC-EVs in mediating the antiinflammatory effects. Mature microglia generated from hiPSCs (iMicroglia) expressed multiple microglia-specific markers. They responded to lipopolysaccharide (LPS) or interferon-gamma challenge by upregulating tumor necrosis factor-alpha (TNF-α) and interleukin-1 beta (IL-1β) mRNA expression and protein release. iMicroglia also exhibited proficiency to phagocytose amyloid-beta (Aβ). The addition of hNSC-EVs decreased TNF-α and IL-1β mRNA expression and the release of TNF-α and IL-1β by LPS-stimulated iMicroglia (proinflammatory human Microglia). However, the antiinflammatory activity of hNSC-EVs on LPS-stimulated microglia was considerably diminished when the PTX3 or miR-21-5p concentration was reduced in EVs. The results demonstrate that hNSC-EVs are proficient for modulating the proinflammatory human microglia into non-inflammatory phenotypes, implying their utility to treat neuroinflammation in neurodegenerative diseases. Furthermore, the role of PTX3 and miR-21-5p in the antiinflammatory activity of hNSC-EVs provides a new avenue for improving the antiinflammatory effects of hNSC-EVs through PTX3 and/or miR-21-5p overexpression.

## Introduction

Extracellular vesicles (EVs) are lipid-bound tiny vesicles with a diameter varying from 30 to 1,000 nm and comprise both smaller exosomes and larger microvesicles (Lötvall et al., 2014; Holm et al., 2018; Vogel et al., 2018; Upadhya and Shetty, 2019, 2021a,b; Vidal, 2019; Upadhya et al., 2020a). In physiological conditions, EVs facilitate the exchange of RNAs and proteins between cells to promote intercellular cross-talk or induce transient or persistent molecular changes in target cells to maintain tissue homeostasis or mediate cellular signaling (Montecalvo et al., 2012; Doyle et al., 2019). On the other hand, EVs shed by different stem cell types can promote specific therapeutic properties, as they carry bioactive microRNAs (miRs), peptides, proteins, and lipids from parental cells. Therefore, to examine their efficacy in treating various diseases, testing the effects of stem cell-derived EV administrations in a variety of preclinical model systems has received significant attention (Kim et al., 2016; Ahn et al., 2021; Go et al., 2021; Xia et al., 2021).

EVs generated from sources such as human mesenchymal stem cells and neural stem cells (hNSCs) have displayed potent neuroprotective, antiinflammatory, and neurogenic properties when tested in cellular assays and animal models of brain disorders (Kim et al., 2016; Long et al., 2017; Gowen et al., 2020; Upadhya et al., 2020b). Moreover, delivering bioactive compounds to sites in and around neurodegeneration or neuroinflammation is likely an essential prerequisite for facilitating brain repair or improving function. In this context, stem cell-derived EVs have received particular attention in treating neurological and neurodegenerative diseases owing to their ability to permeate the entire brain following intranasal (IN) administration (Long et al., 2017; Kodali et al., 2020; Upadhya et al., 2020b). Thus, stem cell-derived EVs are amenable for repeated, non-invasive dispensation as an autologous or allogeneic off-the-shelf product for treating brain disorders. However, a thorough characterization of EVs derived from different stem cell types will be necessary to understand their molecular signatures and potential mechanisms by which they mediate neurorestorative effects before considering stem cell-derived EV therapy for a particular brain condition or disease.

Our recent study has validated that EVs comprising similar miR and protein composition can be isolated consistently from cultured hNSCs derived from human induced pluripotent stem cells (hiPSCs) (Upadhya et al., 2020b). Moreover, such EVs could quickly target most neurons and microglia in virtually all rodent brain regions following an IN administration (Upadhya et al., 2020b). Notably, hNSC-EVs carried miRs and proteins proficient for mediating neuroprotective, anti-apoptotic, antioxidant, blood-brain barrier repairing, neurogenic, and amyloid-beta (Aβ)-reducing properties or promoting synaptogenesis, synaptic plasticity, and better cognitive function. Furthermore, the hNSC-EVs have a cargo of several miRs and proteins capable of mediating antiinflammatory activities. The antiinflammatory properties of hNSC-EVs could be confirmed in an *in vitro* mouse macrophage assay and an *in vivo* mouse status epilepticus-induced acute neuroinflammation model (Upadhya et al., 2020b).

Currently, it is unknown whether hNSC-EVs are proficient in mediating antiinflammatory effects on activated human microglia. Also, the specific proteins and miRs mediating the antiinflammatory effects within hNSC-EVs are yet to be discovered through the knockdown of candidate proteins or miRs that are highly enriched within hNSC-EVs. Therefore, in this study, we first investigated the proficiency of naïve hNSC-EVs to modulate activated human microglia. Testing the effects of hNSC-EVs on human proinflammatory microglia is critical for gaining insights into their potency to modulate activated human microglia in neurodegenerative disease conditions. Then, we probed the role of the protein pentraxin 3 (PTX3) and the miR-21-5p within hNSC-EVs in mediating the antiinflammatory effects. The choice to examine the function of PTX3 and miR-21-5p is based on our previous finding that PTX3 and miR-21-5p are among the most enriched proteins and miRs within hNSC-EVs (Upadhya et al., 2020b). Furthermore, previous studies have shown that PTX3 and miR-21-5p can reduce inflammation, including in models of brain injury and stroke (Sheedy et al., 2010; Barnett et al., 2016; Ge et al., 2016; Shindo et al., 2016; Gaudet et al., 2018; Yang et al., 2018; Rajkovic et al., 2019; Slota and Booth, 2019). Identifying specific molecules that mediate antiinflammatory effects within hNSCs will help comprehend the mechanisms and further improve the antiinflammatory effects of hNSC-EVs through the overexpression of such molecules through transfection of parental stem cells or EV-engineering.

Using standardized protocols, we first generated human microglia from hiPSCs, referred to as iMicroglia, from here onwards. We examined the biological and functional activity of iMicroglia by evaluating their response to lipopolysaccharide (LPS) or interferon-gamma (IFN-γ) challenge and ability to phagocytose fluorescently labeled Aβ particles. Next, we tested the dose-dependent effects of hNSC-EVs to modulate LPS stimulated iMicroglia (i.e., activated or proinflammatory human Microglia). We chose to test hNSC-EVs on LPS activated human iMicroglia because of the contribution of activated microglia in neuroinflammation and brain dysfunction in multiple conditions, including traumatic brain injury, stroke, Parkinson’s disease (PD), and Alzheimer’s disease (AD) (Yun et al., 2018; Lambertsen et al., 2019; Leng and Edison, 2021; Witcher et al., 2021). Then, we tested the efficacy of hNSC-EVs with or without the depletion of PTX3 or miR-21-5p to subdue the LPS stimulated human iMicroglia.

## Materials and Methods

### Generation of iMicroglia From Human Induced Pluripotent Stem Cells

iMicroglia were generated from hiPSCs by following a published protocol (Ijaz et al., 2021) with minor modifications. Briefly, hiPSC colonies (10–16/well) cultured on matrigel-coated dishes using StemFlex medium (Thermo Fisher Scientific, Waltham, MA, United States) were first coaxed into a mesoderm lineage using a basal medium devoid of pluripotency factors but containing bone morphogenetic protein 4 at 80 ng/ml. The basal medium comprised 50 mL of DMEM/F12 containing 1X insulin-transferrin-selenium, 1X non-essential amino acids, 1X Pen-strep, 1X chemically defined lipid concentrate, 64 μg/ml of L-ascorbic acid 2 phosphate sesquimagnesium salt hydrate, and 400 μM of 1-thioglycerol. After 4 days, the cells were driven toward hematopoietic, myeloid, and microglial differentiation by incubating in StemPro-34 SFM™ and components (Thermo Fisher Scientific) supplemented with a combination of cytokines and growth factors at a specified concentration, as described in the published report (Ijaz et al., 2021) for ∼30 days. The floating microglial progenitors were collected and cultured using a microglia maturation medium on poly-d-lysine coated plates for 7–14 days to obtain mature microglia. The media, cytokines and growth factors used in different steps are described in Table 1. Mature iMicroglia were visualized through immunofluorescence using antibodies against several microglia-specific antigens such as integrin alpha M (CD11b), ionized calcium-binding adaptor molecule 1 (IBA-1), and transmembrane protein 119 (TMEM119). The primary antibodies comprised mouse CD11b (1:1,000, Bio-Rad, Hercules, CA, United States), rabbit IBA-1 (1: 1,000, Abcam, Cambridge, MA, United States), and mouse TMEM119 (1:1,000, BioLegend, San Diego, CA, United States). Images of iMicroglia expressing different markers were randomly taken from 3 to 4 independent cultures, and the percentages of total cells (DAPI + cells) expressing CD11b, IBA-1, and TMEM119 were measured. Mature iMicroglia were employed for all subsequent experiments described below.

**TABLE 1:** Media, growth factors and cytokines employed for iMicroglia differentiation from hiPSCs.

Day	Stages	Culture medium	Growth factors and cytokines
−3 to 0	Maintenance of hiPSCs	StemFlex medium	
0–4	Induction into mesoderm lineage	Basal medium	BMP4 (80 ng/mL)
4–6	Differentiation into hematopoietic lineage	StemPro-34 SFM, with supplement and Glutamax (1X)	bFGF (25 ng/mL), SCF (100 ng/mL), VEGF (80 ng/mL)
6–14	Differentiation into Myeloid cells	StemPro-34 SFM, with supplement, and Glutamax (1X)	SCF (50 ng/mL), IL-3 (50 ng/mL), TPO (5 ng/mL), M-CSF (50 ng/mL), Flt3l (50 ng/mL)
14–30	Differentiation into Microglial progenitors	StemPro-34 SFM, with supplement, and Glutamax (1X)	M-CSF (50 ng/mL), Flt3l (50 ng/mL), GM-CSF (25 ng/mL)
30–44	Maturation of Microglia	RPMI 1640 with Glutamax (1X)	GM-CSF (10 ng/mL), IL-34 (100 ng/mL)

*BMP4, Bone Morphogenetic Protein 4; bFGF, Basic fibroblast growth factor; SCF, Stem cell factor; VEGF, Vascular endothelial growth factor; IL-3, Interleukin-3; TPO, Thrombopoietin; M-CSF, Macrophage colony-stimulating factor; Flt3l, FMS-like tyrosine kinase 3 ligand; GM-CSF, Granulocyte-macrophage colony-stimulating factor; IL-34, Interleukin 34.*

### Assessment of Proinflammatory Activity of iMicroglia in Response to Lipopolysaccharide or IFN-γ Stimulation

We first investigated the ability of iMicroglia to sense and respond to the inflammatory stimuli by evaluating their response to the LPS or IFN-γ challenge. For this, iMicroglia were cultured in the microglia maturation medium at a density of 140,000 cells per cm^2^ in 48-well plates and then treated overnight with PBS (vehicle), LPS (100 ng), IFN-γ (50 ng), or LPS + IFN-γ (100 ng + 50 ng). After 24 h, the conditioned media was collected, and the concentration of proinflammatory cytokines, tumor necrosis factor-alpha (TNF-α) and interleukin-1 beta (IL-1β) were measured using ELISA kits (R&D Systems, Minneapolis, MN, United States) with a detection range of 15.6–1,000 pg/mL for TNF-α and 3.9–250 pg/mL for IL-1β.

### Analysis of the Phagocytic Activity of iMicroglia

The ability of iMicroglia to phagocytose fluorescently labeled Aβ particles was investigated, as described elsewhere (Gouwens et al., 2016; Merlo et al., 2018). Briefly, iMicroglia were cultured in the microglia maturation media at a density of 140,000 cells per cm^2^ in 48-well plates and then treated with a fluorescently labeled amyloid-β peptide (FAM-Aβ42; AnaSpec, Fremont, CA, United States) at a concentration of 500 ng/well. The cultures were fixed in 2% paraformaldehyde an hour later and processed for IBA-1 or TMEM119 immunofluorescence. The microglia were imaged to confirm the internalization of FAM-Aβ42 particles into iMicroglia using a fluorescence microscope (Nikon, Melville, NY, United States). The percentages of IBA-1 + /TMEM119 + iMicroglia internalizing FAM-Aβ42 particles were measured from images randomly selected from 3 independent cultures.

### Human Induced Pluripotent Stem Cell-Derived Neural Stem Cell Cultures and Collection of Spent Media for Harvesting Extracellular Vesicles

hNSCs were generated from hiPSCs, as described elsewhere (Yan et al., 2013; Upadhya et al., 2020b). Briefly, hiPSCs (IMR90-4; Wisconsin International Stem Cell Bank, Madison, WI, United States) were expanded into 10–15 colonies in six-well plates coated with matrigel (Corning, Tewksbury, MA, United States) using StemFlex™ medium (Thermo Fisher Scientific, Waltham, MA). The medium was then replaced with the neural induction medium comprising neurobasal (Gibco, Grand Island, New York, United States) and neural induction supplement (Gibco). The media was replaced every day for 10 days, following which primitive NSCs were dissociated with accutase (Gibco) and plated on matrigel-coated dishes with a density of 0.5–1.0 × 10^5^ cells per cm^2^ in an NSC expansion medium containing 50% neurobasal, 50% advanced DMEM/F12, and 1X neural induction supplement. The culture medium was replaced every other day until NSCs reached confluency on day 5 of plating. The NSC cultures were serially passaged when they reached 80% confluency, and NSCs from different passages were cryoprotected and stored in liquid nitrogen. The NSC status at different passages was confirmed through immunofluorescence staining for nestin (anti-nestin, 1:1,000; EMD Millipore, Burlington, MA, United States) and Sox-2 (anti-Sox-2, 1:300; Santacruz Biotechnology, Dallas, TX, United States) as described in our previous study (Upadhya et al., 2020b). In all experiments, the spent media from passage 11 (P11) cultures were collected to isolate hNSC-EVs.

### Isolation of Extracellular Vesicles From Spent Human Induced Pluripotent Stem Cell-Derived Neural Stem Cells Culture Media and Characterization of Extracellular Vesicle Markers

The spent media was centrifuged at low speed to remove cell debris and any suspended particles. The supernatant was passed through a 0.22 μm membrane filter before ultrafiltration to achieve a 5–7-fold concentration using Amicon 100 kDa cut-off UF device (Millipore, Burlington, MA). The EVs were isolated *via* precipitation using overnight incubation with ExoQuick-TC™ at 4^°^C, per the manufacturer’s protocol (System Biosciences, Palo Alto, CA). The EV preparations were centrifuged at 2,000 rpm for 30 min and stored in PBS at −20^°^C. The size analysis of EVs was done using the nanoparticle tracking analysis (NanoSight LM10; Malvern Panalytical, Malvern, United Kingdom), as detailed in our previous studies (Upadhya et al., 2020b). The EVs isolated from hNSC cultures were characterized for EV protein marker expression through western blots (WBs) using antibodies against CD63 (1:1,000 BD Biosciences), CD81 (1:1,000 BD Biosciences), and ALG1-interacting protein X (ALIX, 1:1,000 Santa Cruz).

### Characterization of the Proficiency of Human Induced Pluripotent Stem Cell-Neural Stem Cell Derived-Extracellular Vesicles to Modulate Activated iMicroglia

We first validated the ability of hNSC-EVs to modulate activated iMicroglia. For this, iMicroglia were cultured overnight in the microglia maturation media at a density of 140,000 cells per cm^2^ in 48-well plates. Then, the cultures were incubated with LPS (100 ng/well) and different doses of hNSC-EVs (0, 10, 20, or 40 × 10^9^ EVs) for 24 h, following which the conditioned media and iMicroglia were collected. TNF-α and IL-1β mRNA in iMicroglia were quantified *via* qPCR, and TNF-α and IL-1β protein concentrations in the conditioned media were measured through ELISA, using methods described in our previous reports (Madhu et al., 2019, 2021; Upadhya et al., 2020b).

### Measurement of the Antiinflammatory Protein IL-10

We measured the human IL-10 protein concentration in hNSCs and hNSC-derived EVs. As a positive control, we employed human mesenchymal stem cells (TAMU-IRM generated hMSCs, donor #:6015) as these cells typically express IL-10. The lysates from hNSCs, hMSCs, and hNSC-EVs were prepared using mammalian protein extraction reagent (Thermo Fisher Scientific), and then the ELISA was performed as per manufacturer’s instructions (IL-10 kit from Thermo Fisher Scientific). IL-10 levels were normalized to 1 mg of total protein in the cell or EV lysates.

### Transfection of Human Induced Pluripotent Stem Cell-Derived Neural Stem Cells With siRNA for PTX-3 and Antagomir-21-5p

For generating hNSC-EVs with diminished expression of PTX3 or miR-21-5p, P11 hNSCs with 60% confluency in 6 well plates were transfected with 10 nM siRNA for PTX3 (Santa Cruz, Dallas, TX, United States) or 20 nM miR-21-5p inhibitor (AntagomiR-20-5p, AUM Biotech, Philadelphia, PA, United States) using Lipofectamine™ 2,000 transfection reagent (Invitrogen, Waltham, MA, United States) in Opti-MEM media (Thermo Fisher Scientific). The media was replaced with the NSC medium 5 h later, but the spent media from the first 8 h of incubation after transfection was discarded. The transfected hNSCs were maintained further with new NSC media for 48 h. The spent media from these cultures were used to isolate hNSC-EVs with reduced expression of PTX3 or miR-21-5p, using the method described above.

### Validation of Human Induced Pluripotent Stem Cell-Neural Stem Cell Derived-Extracellular Vesicles With Reduced Expression of PTX3 and miR-21-5p

The reduced expression of PTX3 was first validated in control and transfected hNSCs through measurement PTX3 mRNA *via* qPCR using a specific primer (Qiagen, Germantown, MD, United States) and RT^2^ SYBR Green qPCR Mastermix (Qiagen). Then, PTX3 protein was measured from hNSC-lysates and in EVs isolated from control and transfected hNSCs using sandwich ELISA (Aviscera Biosciences, Sunnyvale, CA, United States). Our previous reports describe the detailed qPCR and ELISA methods employed (Upadhya et al., 2020b; Madhu et al., 2021). Diminished expression of miR-21-5p was validated by measuring miR-21-5p in EVs generated from control and transfected hNSCs, *via* qPCR. For this, the total RNA from hNSC-EVs was first isolated using the SeraMir Exosome RNA amplification kit (System Biosciences), following which miRCURY LNA RT Kit (Qiagen) was employed for converting 5 ng/μl of total RNA into cDNA. miRCURY LNA miRNA SYBR Green PCR kit (Qiagen) and miRCURY LNA miRNA PCR assay primer mix (Qiagen) were employed to measure miR-21–5p level in hNSC-EVs.

### Measurement of Changes in the Antiinflammatory Activity of Human Induced Pluripotent Stem Cell-Neural Stem Cell Derived Extracellular Vesicles With Reduced Expression of PTX3 or miR-21-5p

We investigated whether reduced expression of PTX3 or miR-21-5p in hNSCs would significantly diminish their ability to modulate the activated iMicroglia. iMicroglia were cultured overnight in the microglia maturation media at a density of 140,000 cells per cm^2^ in 48-well plates. Then, the cultures were treated with LPS (100 ng/well), LPS + 40 × 10^9^ naïve hNSC-EVs, LPS + 40 × 10^9^ hNSC-EVs with reduced expression of PTX3, or LPS + 40 × 10^9^EVs hNSC-EVs with reduced expression of miR-21-5p for 24 h. The conditioned media and iMicroglia were collected, TNF-α and IL-1β mRNA in iMicroglia were quantified *via* qPCR, and TNF-α and IL-1β protein concentrations in the conditioned media were measured through ELISA, using methods described in our previous reports (Madhu et al., 2019, 2021; Upadhya et al., 2020b).

### Statistical Analyses

The experiments utilized 3–4 independent cultures per condition, and statistical analysis was done using Prism software 9.0. A two-tailed, unpaired Student’s *t*-test was employed to analyze data across two groups, whereas, one-way ANOVA with Newman-Keuls multiple comparison *post-hoc* tests was employed for datasets containing three or more groups. In all comparisons, *p* < 0.05 was considered a statistically significant value.

## Results

### Mature iMicroglia Generated From Human Induced Pluripotent Stem Cells Displayed Several Microglia-Specific Markers

**FIGURE 1:**
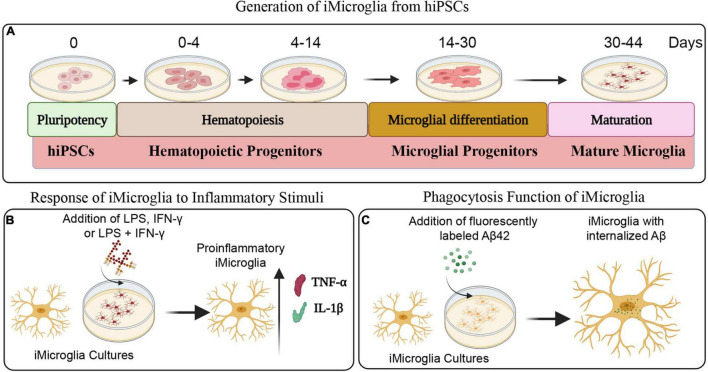
Schematics depicting different steps involved in the generation of iMicroglia from human induced pluripotent stem cells (hiPSCs) and the functional characterization of iMicroglia using specific assays. **(A)** Illustrates the timeline for transforming hiPSCs into mature microglia through stages of hematopoietic progenitor cells and microglial progenitor cells. **(B)** Shows the design employed to generate activated iMicroglia secreting elevated levels of proinflammatory cytokines tumor necrosis factor-alpha (TNF-α) and interleukin-1 beta (IL-1β), using proinflammatory stimuli such as lipopolysaccharide (LPS), interferon-gamma (IFN-γ), or LPS + IFN-γ. **(C)** Depicts the experiment for demonstrating the proficiency of iMicroglia to phagocytose the fluorescently labeled amyloid-beta (Aβ).

**FIGURE 2:**
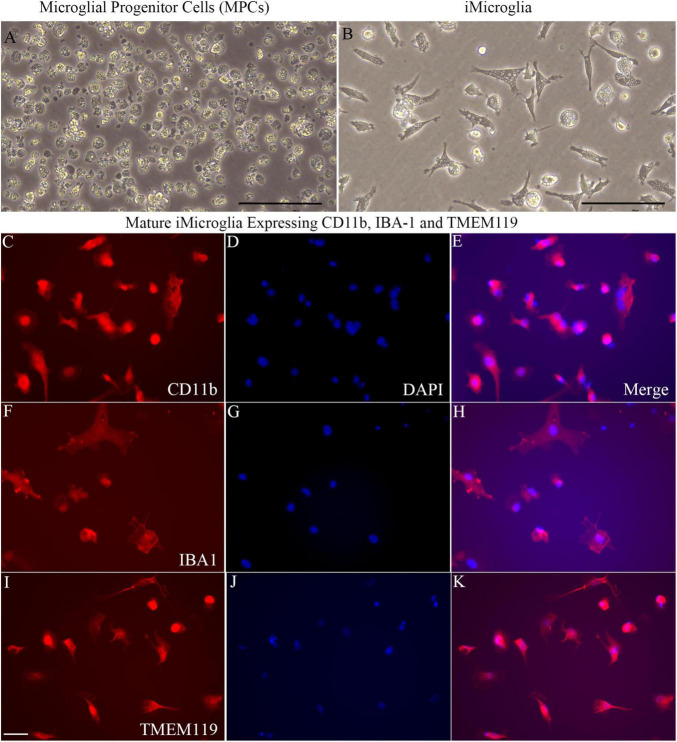
Microglial progenitors from human induced pluripotent stem cells (hiPSCs) differentiate into mature iMicroglia expressing microglia-specific markers. **(A,B)** Illustrate phase contract images of microglial progenitor cells derived from hiPSCs **(A)** and mature iMicroglia **(B)**. **(C–K)** Show the morphology of mature iMicroglia expressing microglia-specific markers such as CD11b **(C–E)**, IBA-1 **(F–H)**, and TMEM119 **(I–K)**. Scale bar **(A,B)** 100 μm; **(C–K)** 25 μm.

**FIGURE 3:**
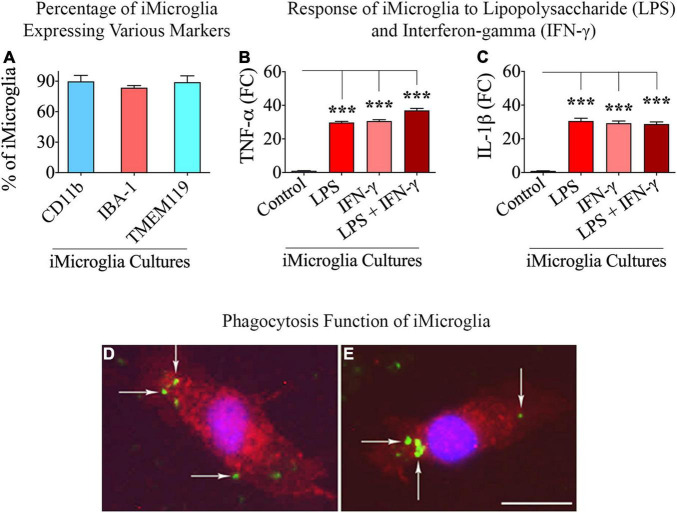
Percentages of total cells expressing microglia-specific markers in iMicroglia cultures, the response of iMicroglia to proinflammatory stimuli, and phagocytosing property of iMicroglia. Bar chart **(A)** shows percentages of total cells expressing CD11b, IBA-1, and TMEM119 in iMicroglia cultures. The bar charts in **(B,C)** show iMicroglia cultures activated through lipopolysaccharide (LPS), interferon-gamma (IFN-γ), or LPS + IFN-γ, secreting elevated levels of proinflammatory cytokines tumor necrosis factor-alpha (TNF-α) and interleukin-1 beta (IL-1β). **(D,E)** Illustrate mature IBA-1 + iMicroglia (red) following the internalization of fluorescently labeled Aβ (green), confirming their proficiency for phagocytosis. ***p < 0.001, **(D,E)** 25 μm.

Figure 1 illustrates the various stages in the differentiation of hiPSCs into mature iMicroglia (Figure 1A), and the steps involved in testing the proinflammatory response of mature iMicroglia with LPS and/or IFN-γ stimulation (Figure 1B), and the phagocytic property of mature iMicroglia (Figure 1C). The iMicroglia differentiation protocol employed in the study (Ijaz et al., 2021) efficiently transformed hiPSCs into mature microglia on days 37–44 after passing through stages of myeloid and hematopoietic progenitor cells (HPCs) on days 4–12 and microglia progenitor cells (MPCs) on days 30–36 (Figures 2A,B). Phase-contrast pictures of MPCs and mature iMicroglia are illustrated in Figures 2A,B. We next probed whether iMicroglia generated from hiPSCs express the three typical markers of mature microglia through immunofluorescence. The analysis confirmed that most MPCs maintained in the microglia maturation medium expressed CD11b, IBA-1, and TMEM119 (Figures 2C–K). Quantification revealed that ∼84–89% of total cells expressed CD11b (Mean ± S.E.M. = 89.8 ± 5.9%), IBA-1 (83.7 ± 2.1%) and TMEM119 (89.1 ± 6.3%), implying the proficiency of the employed protocol for transforming hiPSCs into mature iMicroglia (Figure 3A).

### Mature iMicroglia From Human Induced Pluripotent Stem Cells Displayed Proinflammatory Activity in Response to Lipopolysaccharide or IFN-γ

We investigated the ability of mature iMicroglia to display proinflammatory activity in response to treatment with the bacterial endotoxin LPS or the cytokine IFN-γ, alone or in combination. iMicroglia treated with LPS, IFN-γ, or LPS + IFN-γ responded similarly by greatly enhancing the release of proinflammatory cytokines TNF-α and IL-1β into the media (Figures 3B,C). In comparison to the vehicle-treated iMicroglia, the release of TNF-α and IL-1β by LPS and/or IFN-γ treated iMicroglia was increased by 29–37 folds (*p* < 0.001, Figures 3B,C), implying that iMicroglia can readily transform themselves into a proinflammatory phenotype when faced with the pathogens or the proinflammatory stimulus.

### Mature iMicroglia From Human Induced Pluripotent Stem Cells Are Proficient in Phagocytosing Aβ

We examined whether mature iMicroglia are capable of phagocytosing proteins such as Aβ. Incubation of mature iMicroglia with fluorescently labeled Aβ (i.e., FAM-Aβ42) for an hour resulted in the internalization of Aβ by iMicroglia. Figures 3D,E illustrates examples of iMicroglia displaying internalized FAM-Aβ42 particles, implying their proficiency for phagocytosing unfolded proteins. Quantification revealed that 71.0 ± 5.2% of IBA-1 + iMicroglia and 68.5 ± 4.7% of TMEM119 iMicroglia internalized FAM-Aβ42 particles with an hour of incubation.

### Size and Extracellular Vesicle-Specific Marker Expression in Human Induced Pluripotent Stem Cell-Neural Stem Cell Derived Extracellular Vesicles

**FIGURE 4:**
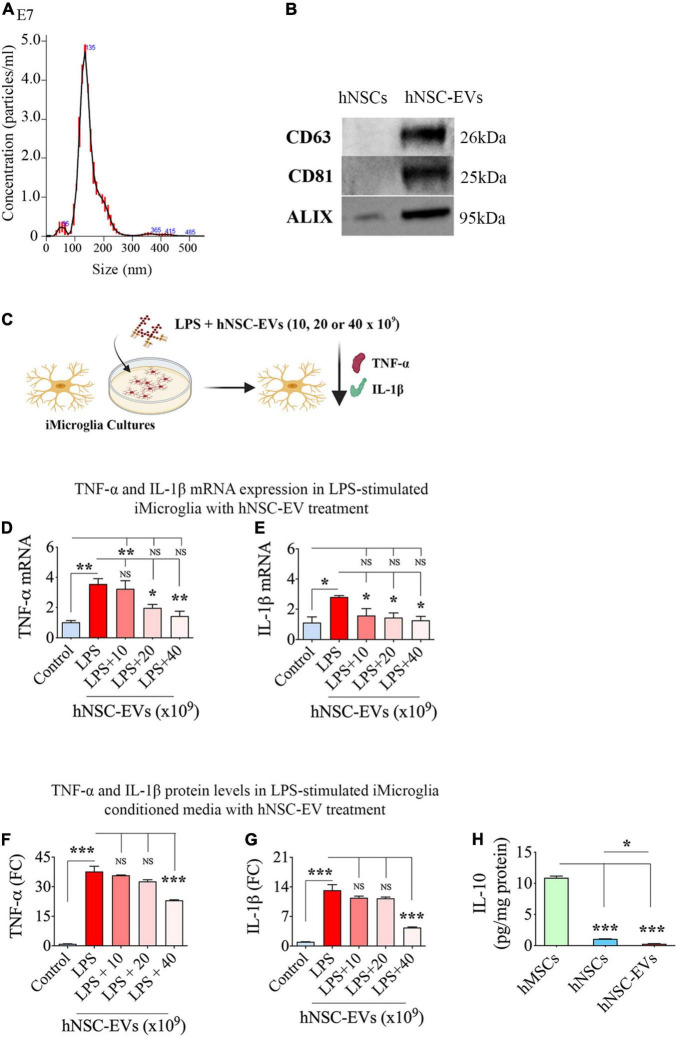
Size and marker expression in human neural stem cell-derived extracellular vesicles (hNSC-EVs) and antiinflammatory activity of hNSC-EVs. **(A)** Shows the size of hNSC-EVs measured through nanoparticle tracking analysis. **(B)** Illustrates western blot images of EV-specific markers CD63, CD81, and ALIX in hNSCs and hNSC-EVs. **(C)** Is a schematic showing the experimental design testing the antiinflammatory effect of hNSC-EVs. The bar charts in **(D,E)** show greatly enhanced tumor necrosis factor-alpha (TNF-α) and interleukin-1 beta (IL-1β) mRNA expression in iMicroglia with lipopolysaccharide (LPS) treatment alone and diminished TNF-α and IL-1β mRNA expression in iMicroglia with LPS plus hNSC-EV treatment. While only higher doses of hNSC-EV treatment (20 or 40 × 10^9^ EVs) normalized TNF-α mRNA expression **(D)**, all doses of hNSC-EV treatment tested (10, 20, or 40 × 10^9^ EVs) normalized IL-1β mRNA expression **(E)**. The bar charts in **(F,G)** show elevated TNF-α, and IL-1β protein levels in LPS-stimulated iMicroglia conditioned media and significantly diminished TNF-α and IL-1β protein concentrations in the conditioned media of LPS-stimulated iMicroglia receiving 40 × 10^9^ EVs treatment. **(H)** Shows the concentration of IL-10 in hNSCs and hNSC-EVs compared to human mesenchymal stem cells (hMSCs). *p < 0.05, **p < 0.01, ***p < 0.001, NS, not significant.

The size of EVs isolated through ExoQuick-TC™ was measured using nanoparticle tracking analysis. Such quantification revealed a mode value of 133.3 ± 1.0 nm for EVs (Figure 4A), indicating the separation of smaller EVs. Furthermore, WB analysis of hNSC-EVs confirmed the presence of EV-specific markers such as CD63, CD81, and ALIX (Figure 4B).

### Human Induced Pluripotent Stem Cell-Neural Stem Cell Derived Extracellular Vesicle Treatment Modulated the Release of TNF-α and IL-1β by Proinflammatory iMicroglia

We validated the ability of hNSC-EVs to modulate activated iMicroglia by treating mature iMicroglia cultures with LPS and different doses of hNSC-EVs (0, 10, 20, or 40 × 10^9^ EVs) for 24 h and then measuring changes in TNF-α and IL-1β mRNA expression in iMicroglia, and TNF-α and IL-1β protein levels in the medium (Figure 4C). LPS treatment alone increased TNF-α mRNA expression in iMicroglia by ∼3.5 folds (*p* < 0.01, Figure 4D). The addition of hNSC-EVs dose-dependently decreased TNF-α mRNA expression in iMicroglia. While the lowest EV dose (i.e., 10 × 10^9^ EVs) had no effect, higher EV doses were efficient in curbing the increase of TNF-α mRNA expression in LPS stimulated iMicroglia (*p* < 0.01–0.05 vs. LPS alone and *p* > 0.05 vs. control), with the highest dose (i.e., 40 × 10^9^ EVs) mediating the most potent antiinflammatory activity (60% reduction vs. LPS alone, *p* < 0.01, Figure 4D). LPS treatment alone increased IL-1β mRNA expression in iMicroglia by ∼2.5 folds (Figure 4E). The addition of hNSC-EVs decreased IL-1β mRNA expression in iMicroglia with all doses (10, 20, or 40 × 10^9^ EVs) exhibiting significant antiinflammatory activity (44–55% reduction vs. LPS alone, *p* < 0.05, Figure 4E). Also, IL-1β mRNA expression in LPS stimulated iMicroglia restored to levels in naïve control iMicroglia with the addition of all doses of hNSC-EVs (*p* > 0.05). Quantification of TNF-α and IL-1β protein levels in iMicroglia culture media revealed that LPS stimulation alone enhanced TNF-α release by ∼38 folds and IL-1β by ∼13-fold (*p* < 0.001, Figures 4F,G). The highest dose of EV treatment considerably reduced the release of both TNF-α and IL-1β by LPS stimulated iMicroglia (39–67% reduction vs. LPS alone, *p* < 0.001, Figures 4F,G). Thus, an apt dose of hNSC-EVs can considerably suppress the proinflammatory activity of human microglia.

To determine the possible involvement of the antiinflammatory protein IL-10 in the antiinflammatory effects of hNSC-EVs, we measured the IL-10 concentration in hNSCs and hNSC-EVs. We also measured IL-10 from hMSCs as a positive control since these MSCs are known to produce IL-10 (Heo et al., 2016; Al-Azzawi et al., 2020). Our results showed that both hNSCs and hNSC-EVs contained only negligible amounts of IL-10 compared to hMSCs (Figure 4H), implying that IL-10 is not directly involved in the antiinflammatory effects mediated by hNSC-EVs.

### Human Induced Pluripotent Stem Cell-Neural Stem Cell Derived-Extracellular Vesicles With Reduced Expression of PTX3 Mediated Diminished Antiinflammatory Activity on Lipopolysaccharide Stimulated iMicroglia

**FIGURE 5:**
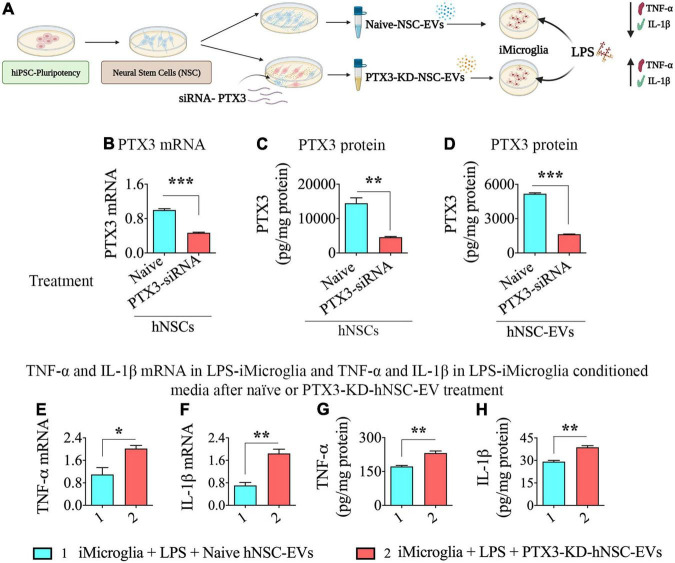
Human neural stem cell-derived extracellular vesicle (hNSC-EVs) with reduced expression of pentraxin 3 (PTX3) mediated diminished antiinflammatory activity on lipopolysaccharide (LPS) stimulated iMicroglia. **(A)** Is a schematic showing the design for this experiment. The bar charts in **(B–D)** show reductions in PTX3 mRNA expression in hNSCs **(B)**, PTX3 protein concentration in the hNSC lysate, and PTX3 protein concentration in hNSC-EVs **(C)** after PTX3-siRNA treatment. The bar charts in **(E,F)** illustrate that LPS-stimulated iMicroglia display higher expression of tumor necrosis factor-alpha (TNF-α; **E**) and interleukin-1 beta (IL-1β; F) mRNA expression when treated with hNSC-EVs with reduced expression of PTX3 (PTX3-knockdown [KD]-hNSC-EVs), in comparison to naïve hNSC-EVs. The bar charts in **(G,H)** show that LPS-stimulated iMicroglia release higher levels of TNF-α **(G)** and IL-1β **(H)** into the culture media when treated with hNSC-EVs with PTX3-KD-hNSC-EVs, in comparison to naïve hNSC-EVs. *p < 0.05; **p < 0.01; ***p < 0.001.

The cartoon in Figure 5A illustrates the various steps involved in this experiment. We first validated the efficiency of PTX3-siRNA to significantly reduce PTX3 mRNA expression in transfected hNSCs with the employed protocol. In comparison to naïve hNSCs, the transfected hNSCs displayed a 53% decline in PTX3 mRNA expression (*p* < 0.001, Figure 5B), and the PTX3 protein was reduced by 68% in the transfected hNSCs (*p* < 0.01, Figure 5C). Importantly, in EVs isolated from transfected hNSC conditioned media, the PTX3 protein level was decreased by 68% compared to EVs from naïve hNSCs (*p* < 0.0.001, Figure 5D). Thus, PTX3-siRNA treatment to hNSCs did not result in complete knockdown (KD) of PTX3 but significantly reduced PTX3 mRNA expression in hNSCs and PTX3 protein concentration in hNSC-derived EVs.

Next, we added naïve hNSC-EVs or hNSC-EVs with reduced expression of PTX3 (PTX3-KD-hNSC-EVs) to LPS treated iMicroglia, incubated for 24 h, and measured TNF-α and IL-1β mRNA expression in iMicroglia, and TNF-α and IL-1β protein levels in the conditional media. The dose of naïve hNSC-EVs (40 × 10^9^ EVs) exhibiting robust antiinflammatory activity in the previous experiment (i.e., dose-response effects of hNSC-EVs on LPS or IFN-γ stimulated iMicroglia, Figure 4) was employed. LPS stimulated iMicroglia displayed higher levels of both TNF-α and IL-1β mRNA expression with the addition PTX3-KD-hNSC-EVs, in comparison to the addition of naïve hNSC-EVs (*p* < 0.01–0.05, Figures 5E,F). Moreover, both TNF-α and IL-1β protein levels in the conditioned media of LPS stimulated iMicroglia were increased with the addition PTX3-KD-hNSC-EVs, in comparison to the addition of naïve hNSC-EVs (*p* < 0.01, Figures 5G,H). Thus, the antiinflammatory activity hNSC-EVs on LPS stimulated iMicroglia significantly diminished when PTX3 concentration in EVs was decreased, implying the critical role played by PTX3 in hNSC-EV mediated antiinflammatory activity on activated human microglia.

### Human Induced Pluripotent Stem Cell-Neural Stem Cell Derived Extracellular Vesicles With Reduced Expression of miR-21-5p Mediated Diminished Antiinflammatory Activity on Lipopolysaccharide Stimulated iMicroglia

**FIGURE 6:**
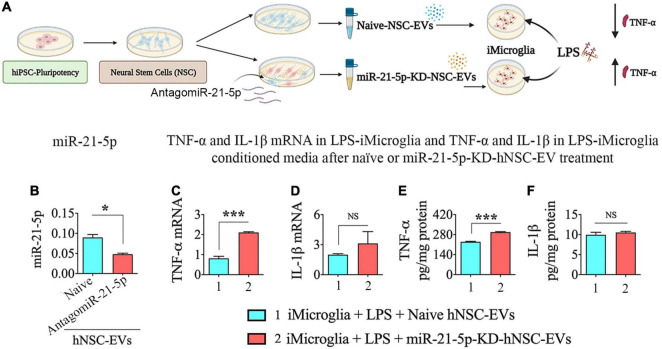
Human neural stem cell-derived extracellular vesicle (hNSC-EVs) with reduced expression of miR-21-5p mediated diminished antiinflammatory activity on lipopolysaccharide (LPS) stimulated iMicroglia. **(A)** Is a schematic depicting the experimental design. The bar chart **(B)** shows the reduction in miR-21-5p content of hNSC-EVs **(C)** after antagomiR-21-5p treatment. The bar charts in **(C,D)** show that LPS-stimulated iMicroglia display enhanced expression of tumor necrosis factor-alpha (TNF-α; **C**) expression with no significant change in interleukin-1 beta (IL-1β; **D**) mRNA expression when treated with hNSC-EVs with reduced expression of miR-21-5p (miR-21-5p-knockdown [KD]-hNSC-EVs), in comparison to naïve hNSC-EVs. The bar charts in **(E,F)** show that LPS-stimulated iMicroglia release a higher concentration of TNF-α **(E)** with no change in IL-1β concentration **(F)** into the culture media when treated with hNSC-EVs with miR-21-5p-KD-hNSC-EVs, in comparison to naïve hNSC-EVs. *p < 0.05; ***p < 0.001; NS, not significant.

The cartoon in Figure 6A illustrates the various steps involved in this experiment. We first confirmed the efficiency of antagomiR-21-5p to significantly reduce miR-21-5p expression in EVs isolated from transfected hNSCs in the employed protocol (Figure 6B). The EVs isolated from transfected hNSCs displayed a 46% decline in miR-21-5p expression in comparison to EVs from naïve hNSCs (*p* < 0.05, Figure 6B). Thus, the miR-21-5p-siRNA treatment protocol to hNSCs employed in the study did not eliminate miR-21-5p but significantly reduced miR-21-5p in the cargo of hNSC-derived EVs.

Next, we added naïve hNSC-EVs or hNSC-EVs with reduced expression of miR-21-5p (miR-21-5p-KD-hNSC-EVs) to LPS treated iMicroglia, incubated for 24 h, and measured TNF-α and IL-1β mRNA expression in iMicroglia, and TNF-α and IL-1β protein levels in the conditioned medium. LPS stimulated iMicroglia displayed a higher level of TNF-α mRNA expression with the addition miR-21-5p-KD-hNSC-EVs, in comparison to the addition of naïve hNSC-EVs (*p* < 0.001, Figure 6C). However, the IL-1β mRNA expression in LPS stimulated iMicroglia was similar with naïve hNSC-EV or miR-21-5p-KD-hNSC-EV treatment (*p* > 0.05, Figure 6D). Measurement of TNF-α and IL-1β protein levels in the conditioned medium of LPS stimulated iMicroglia also showed a similar trend. The conditioned medium from LPS stimulated iMicroglia displayed a higher level of TNF-α protein with the addition miR-21-5p-KD-hNSC-EVs, in comparison to the addition of naïve hNSC-EVs (*p* < 0.001, Figure 6E). However, the IL-1β protein concentrations in the conditioned media of LPS stimulated iMicroglia were similar with naïve hNSC-EV or miR-21-5p-KD-hNSC-EV treatment (*p* > 0.05, Figure 6F). Thus, the antiinflammatory activity hNSC-EVs on LPS stimulated iMicroglia partially diminished when miR-21-5p content was decreased in EVs, implying that miR-21-5p also plays a role in hNSC-EV mediated antiinflammatory activity. The differential effect of miR-21-5p-KD-hNSC-EVs on TNF-α and IL-1β expression and release by LPS stimulated iMicroglia likely reflects the effect of a partial KD of miR-21-5p in hNSC-EVs.

## Discussion

This study demonstrates that appropriate doses of hNSC-EV treatment can modulate proinflammatory human microglia into non-inflammatory phenotypes, and PTX3 and miR-21-5p are among the EV-cargo involved in the antiinflammatory activity of hNSC-EVs. The therapeutic effects of hNSC-EVs on activated human microglia was evident from decreased TNF-α and IL-1β mRNA expression and diminished release of TNF-α and IL-1β by LPS-stimulated iMicroglia in this study. The involvement of PTX3 in the antiinflammatory activity of hNSC-EVs on activated human microglia was apparent from higher levels of TNF-α and IL-1β mRNA expression and TNF-α and IL-1β protein release by LPS-stimulated iMicroglia when they were treated with hNSC-EVs with reduced expression of PTX3 vis-à-vis the addition of naïve hNSC-EVs. On the other hand, the involvement of miR-21-5p in the antiinflammatory activity of hNSC-EVs on activated human microglia seemed partial because the addition of hNSC-EVs with reduced miR-21-5p resulted in higher levels of TNF-α mRNA and protein levels but not IL-1β mRNA and protein concentrations.

The antiinflammatory activity of hNSC-EVs was first observed on LPS stimulated mouse macrophages using a cell culture assay in our previous study (Upadhya et al., 2020b). In the latter study, LPS exposure alone induced elevated release of IL-6 from macrophages. However, the addition of hNSC-EVs to LPS-stimulated macrophages demonstrated dose-dependent antiinflammatory effects with 4 × 10^9^ hNSC-EVs or higher, significantly suppressing the release of IL-6 from activated macrophages (Upadhya et al., 2020b). Then, in a mouse model of acute seizures, brain injury, and neuroinflammation (i.e., a prototype of status epilepticus), IN administration of hNSC-EVs 2 h after the induction of status epilepticus normalized the concentration of multiple proinflammatory cytokines involved in post-status epilepticus cytokine storm in the hippocampus (Upadhya et al., 2020b). The normalized cytokines included TNF-α, IL-1β, IFN-γ, and monocyte chemoattractant protein-1. Interestingly, such treatment also normalized the antiinflammatory cytokine IL-10. The above results provided evidence on the ability of hNSC-EVs to modulate the inflammatory activity of activated macrophages *in vitro* or neuroinflammatory changes that transpire in the adult rodent brain after an insult. Nonetheless, it remained to be addressed whether hNSC-EVs are proficient in mediating antiinflammatory effects on activated human microglia.

Testing the effects of hNSC-EVs on human proinflammatory microglia is critical for gaining insights into their potency to modulate activated human microglia in neurodegenerative disease conditions. For example, it has been proposed that functional changes occurring in microglia are vital factors in the pathogenesis of AD and PD (Zhang et al., 2013; Mhatre et al., 2015; Salter and Stevens, 2017; Subramaniam and Federoff, 2017). Increased microglial activation has also been hypothesized to contribute to neuronal dysfunction and death in such diseases (Svoboda et al., 2019). However, activated human microglia associated with neurodegenerative diseases are not readily available for extensive studies, likely due to technical and logistical obstacles in harvesting pure, viable microglia from postmortem brain tissues of subjects with neurodegenerative diseases (Olah et al., 2018; Mathys et al., 2019; Alsema et al., 2020). Also, microglia are very sensitive to culture conditions and could lose their key *in situ* properties when grown *in vitro* (Svoboda et al., 2019).

Therefore, we directed our studies on iMicroglia generated from hiPSCs, understanding that antiinflammatory studies need biologically and functionally active iMicroglia. By employing a published protocol with a few modifications (Ijaz et al., 2021), we were able to generate iMicroglia from hiPSCs, which comprised induction of hiPSCs first into a hematopoietic lineage and then into myeloid intermediates or primitive macrophages (Muffat et al., 2016; Abud et al., 2017; Douvaras et al., 2017; Pandya et al., 2017). Since differentiation of microglia *in vivo* is regulated by cytokines released from neurons and astrocytes (Erblich et al., 2011; Wang et al., 2012), microglial differentiation *in vitro* was accomplished by adding such cytokines. Our studies demonstrated that iMicroglia generated through such a protocol expressed microglia-specific markers such as CD11b, IBA-1, and TMEM119. In addition, iMicroglia efficiently responded to proinflammatory stimuli from LPS and IFN-γ and most iMicroglia exhibited proficiency for phagocytosing unfolded proteins such as Aβ. The generation of such iMicroglia facilitated the investigation of the direct effects of hNSC-EVs on activated human microglia in this study. Our results showing that appropriate doses of hNSC-EV treatment can modulate the release of proinflammatory cytokines TNF-α and IL-1β by activated human microglia suggests that such EVs could be used for treating neuroinflammation in neurodegenerative diseases. However, studies testing the effects of hNSC-EVs on iMicroglia produced from hiPSCs of patients afflicted with different neurodegenerative diseases are needed in the future to advance the concept of hNSC-EV therapy for inflammatory microglia. This is because iMicroglia produced from patient-iPSCs likely display disease-specific pathological changes. For example, a study has shown that iMicroglia produced from sporadic AD-patient iPSCs display altered phagocytosis and release elevated levels of specific cytokines with the LPS challenge (Xu et al., 2019).

The antiinflammatory activity of hNSC-EVs likely comprises synergized effects of several proteins and miRs in their cargo. Therefore, identifying specific molecules within hNSC-EVs mediating antiinflammatory effects is beneficial in comprehending the mechanisms and further improving the antiinflammatory effects of hNSC-EVs through the overexpression of such molecules in EVs through transfection of parental cells or EV-engineering approaches. To address this issue, we initiated such studies by investigating the role of PTX3 and miR-21-5p in the antiinflammatory activity of hNSC-EVs. Notably, we found a significantly diminished antiinflammatory activity of hNSC-EVs on LPS stimulated iMicroglia when PTX3 concentration in hNSC-EVs was reduced by 68% through treatment of hNSCs with PTX3-siRNA. The antiinflammatory activity of hNSC-EVs on activated iMicroglia was also partially reduced when miR-21-5p content in hNSC-EVs was decreased by 46% by treating hNSCs with antagomiR-21-5p. These results implied that PTX3 and miR-21-5p play vital roles in the antiinflammatory activity mediated by hNSC-EVs.

In addition to being one of the highly enriched proteins in hNSC-EVs (Upadhya et al., 2020b), there were additional reasons for investigating the role of PTX3 in the antiinflammatory effects, which are summarized below. PTX3 has been shown to promote neuroprotection, neurogenesis, and angiogenesis after ischemic brain injury (Rodriguez-Grande et al., 2015). A compromised resolution of brain edema and glial scar formation was observed after ischemic brain injury in PTX3 deficient mice (Rodriguez-Grande et al., 2014, 2015), and astrocyte-derived PTX3 maintained blood-brain barrier integrity after stroke (Shindo et al., 2016, 2021). Additional studies have shown that PTX3 treatment after traumatic brain injury can activate the beneficial type 2 astrocytes, enhance neuroprotection and neurogenesis, and improve functional recovery (Zhou et al., 2020). PTX3 can regulate neutrophil transmigration into the brain in neuroinflammatory conditions (Rajkovic et al., 2019), and PTX3 secreted by human adipose tissue-derived stem cells can reduce dopaminergic neurodegeneration and promote functional recovery in a model of PD (Lian et al., 2021). PTX3 also plays a role in synaptogenesis in the developing brain (Fossati et al., 2019). Furthermore, PTX3 deficiency results in augmented infarct area following myocardial ischemia/reperfusion injury (Salio et al., 2008), increased macrophage accumulation and inflammation in atherosclerotic plaques (Norata et al., 2009), and exacerbated proinflammatory gene expression in visceral adipose tissue from a high fat diet-induced obese mice (Guo et al., 2020). Overall, PTX3 can mediate multiple beneficial effects in various disease states through antiinflammatory and neuroprotective actions. The current study has further demonstrated that PTX3 within hNSC-EVs is indeed involved in modulating human proinflammatory microglia into a non-inflammatory phenotype. Thus, PTX3 appears to be an excellent candidate to overexpress in hNSC-EVs to enhance their therapeutic efficacy in neurodegenerative and neuroinflammatory conditions.

The investigation of miR-21-5p, one of the most enriched miRs in hNSC-EVs, in the antiinflammatory activity of hNSC-EVs was also based on multiple beneficial effects of this miR observed in several previous studies. For example, age-related cognitive dysfunction is associated with dysregulation of miR21 (Sessa et al., 2019). In a cell culture model of traumatic brain injury, miR-21 has been shown to suppress the activation of caspase-3 and alleviate apoptosis of cortical neurons (Han et al., 2014). Another study has shown that miR-21 can alleviate the injured brain microvascular endothelial barrier leakage by suppressing inflammation and apoptosis by regulating NF-kB signaling (Ge et al., 2016). miR-21 expression is also upregulated in the surviving neurons adjoining infarcts in a stroke model, implying its role in neuroprotection (Buller et al., 2010). miR-21 has also been shown to mediate antiinflammatory effects in a model of ischemic stroke (Gaudet et al., 2018). miR-21 can mediate antiinflammatory effects through multiple mechanisms, which include downregulation of NF-kB, induction of the antiinflammatory cytokine IL-10, and directly decreasing the release of TNF-α (Sheedy et al., 2010; Das et al., 2014; Barnett et al., 2016; Slota and Booth, 2019). miR-21 has also been shown to attenuate inflammation, cardiac dysfunction, and maladaptive remodeling after myocardial infarction (Yang et al., 2018; Li et al., 2021). Taken together, it is clear that miR-21 can mediate robust antiinflammatory in a variety of brain and heart conditions.

The current results demonstrate that miR-21 within hNSC-EVs also contributes to suppressing human proinflammatory microglia, particularly the release of TNF-α. However, downregulation of miR-21 in hNSC-EVs did not impact IL-1β released from proinflammatory microglia. The discrepancy regarding the effects of miR-21 downregulation on TNF-α and IL-1β release by proinflammatory microglia is likely due to the following factors. miR-21 can directly reduce TNF-α concentration in microglia by inhibiting glycogen synthase kinase-b (GSK3b) through its effect on phosphatase and tensin homolog (Steinbrecher et al., 2005; Das et al., 2014). On the other hand, in the presence of miR-21, other proinflammatory cytokines such as IL-1β can persist as IL-1β release could be maintained by other changes in activated microglia such as the activation of NOD-, LRR- and pyrin domain-containing protein 3 (NLRP3) inflammasomes (Heneka et al., 2018; Madhu et al., 2021). Nonetheless, miR-21 could be another candidate to overexpress in hNSC-EVs to improve their therapeutic efficacy in neurodegenerative and neuroinflammatory conditions because of its ability to reduce TNF-α concentration.

In conclusion, the results underscore that hNSC-EV treatment can modulate proinflammatory human microglia into non-inflammatory phenotypes, and PTX3 or miR-21-5p are among the EV-cargo involved in the antiinflammatory activity of hNSC-EVs. Studies testing the effects of hNSC-EVs overexpressing PTX3 and miR-21, alone or in combination, in AD, PD, or other disease models in the future can determine the specific benefits of such strategies.

## Data Availability Statement

The original contributions presented in the study are included in the article/supplementary material, further inquiries can be directed to the corresponding author/s.

## Author Contributions

AS: concept and finalization of manuscript text and figures. RU, LM, and SR: generation and characterization of iMicroglia from hiPSCs, isolation and characterization hNSC-EVs, data collection and analysis, and assays using iMicroglia. LM: biochemical and qPCR measurements. RU, LM, and AS: interpretation of data and preparation of figures. RU and LM: first draft of the manuscript. All authors provided feedback and edits to the manuscript text and approved the final version.

## Conflict of Interest

The authors declare that the research was conducted in the absence of any commercial or financial relationships that could be construed as a potential conflict of interest.

## Publisher’s Note

All claims expressed in this article are solely those of the authors and do not necessarily represent those of their affiliated organizations, or those of the publisher, the editors and the reviewers. Any product that may be evaluated in this article, or claim that may be made by its manufacturer, is not guaranteed or endorsed by the publisher.
